# Dataset: local government mask orders preceding statewide orders by US states

**DOI:** 10.12688/f1000research.27614.1

**Published:** 2021-01-08

**Authors:** Philip Jacobs, Arvi Ohinmaa

**Affiliations:** 1 Department of Medicine, University of Alberta, Edmonton, Alberta, T5T 2W8, Canada

**Keywords:** City mask orders, County mask orders, COVID-19 masks, local government prevention

## Abstract

We present a database listing local government mask orders for COVID-19 that were enacted between April and September, 2020, prior to the date that the governors issued statewide mask wearing mandates. We obtained data from a Google search of web pages of local and national commercial and public broadcasters and newspapers, and of the orders themselves.  In the database, we present data identifying the county, municipality or tribal council, date of the order, and the source’s internet address. In the 34 states with statewide orders, local governments in 21 of these states issued mandates in 218 municipalities, 155 counties, and 1 tribal council.  The dataset can be accessed from https://doi.org/10.7939/DVN/NDFEHK

## Introduction

During the Spring of 2020, the use of face masks in public places emerged as an important determinant of the prevention of COVID-19. By August, 2020 public health officers in 34 US states had issued statewide orders for occupants to wear masks in public places ^1^. In many of these states, local governments issued their own mask orders prior to the statewide orders. When we are considering the impact of mask wearing orders, we need to know the full extent to which local governments required occupants to wear masks in public. We developed a dataset of mask orders by local government units (counties and cities) in the states which eventually enacted statewide orders, and the dates which these orders came into effect.

## Methods

Our initial sample consisted of 34 states whose governments issued statewide mask wearing mandates by 1 September, 2020. Starting with the date that each state issued statewide orders, and going backwards until early April, we conducted Google searches with the following search terms: state AND city or county or tribal group (general and specific terms) AND COVID-19 AND “mask order” or “mask mandate” AND date (backwards from state order date). From the resulting articles we searched first for website news articles from local newspapers, commercial TV and radio stations, and local Public Radio (NPR) or television (PBS) stations that listed government mask orders. If there was no statewide list, we then searched for articles on orders from key counties and cities in all of the remaining states. From these items, and for each state, we developed a list of cities and counties where orders had been reported. We recorded the date on which each order came into effect, and also the internet address of the mask order or news source reporting on a mask order. 

## Dataset description

Among the 34 states that issued statewide orders, counties, cities or tribal councils in 21 states issued orders prior to the statewide mandates in 21 states. We could not find any early local orders in the following 13 states: Connecticut, Delaware, Hawaii, Kentucky, Maine, Maryland, Nevada, New Jersey, New Mexico, New York, Pennsylvania, Virginia, and West Virginia. In the accompanying Excel file ( *Underlying data*), we present the state name, the name of the local area, the designation of the area as a county (C), municipality (M) or Tribal Council (T), and the date the local mask order came in effect and the reference for the mask order.

We present data on the number of orders by C, M, and T, along with the date of the state order going into effect in Table 1.

**Table 1.** Local government mask orders preceding statewide orders by US states.

State	Statewide order date	Early county mandates	Early municipal mandates	Early tribal mandates
Alabama	16-Jul-20	2	7	0
Arkansas	20-Jul-20	0	11	0
California	18-Jun-20	35	22	0
Colorado	17-Jul-20	13	22	0
Illinois	1-May-20	0	20	0
Indiana	27-Jul-20	7	4	0
Kansas	3-Jul-20	2	0	0
Louisiana	13-Jul-20	2	2	0
Massachusetts	6-May-20	0	51	0
Minnesota	25-Jul-20	0	12	0
Mississippi	4-Aug-20	37	0	0
Montana	15-Jul-20	1	2	1
North Carolina	26-Jun-20	3	4	0
Ohio	23-Jul-20	21	23	0
Oregon	1-Jul-20	7	0	0
Rhode Island	8-May-20	0	1	0
Texas	3-Jul-20	13	23	0
Vermont	1-Aug-20	0	7	0
Washington	26-Jun-20	2	0	0
Wisconsin	1-Aug-20	4	7	0
Michigan	27-Apr-20	1	0	0

We should note that although we list Mississippi state as having local mask orders, in fact it was the Governor who issued the counties’ orders: counties were exempt from the orders if they had incidences of COVID-19 below rates set by the Governor’s office and the State Health Officer.

## Summary

Our dataset shows the number of local government units that established mask orders prior to the states issuing statewide orders. In Table 1 we show the number of local government orders for each state. In the 34 states, 218 municipalities, 155 counties and 1 tribal council issued orders.

## Data availability

University of Alberta Library Dataverse: Local mask orders pre Statewide, https://doi.org/10.7939/DVN/NDFEHK ^2^.

The database contains detailed collected data for 21 states with local orders that were in effect prior to statewide orders:

A. County, Municipality or Tribal Council
B. State
C. Identification of locality as county (C), City or town (M), or Tribal Council (T) + source data embedded.
D. Date the local order came into effect

Data are available under the terms of the Creative Commons Zero “No rights reserved” data waiver (CC0 1.0 Public domain dedication).
