# Supplementary material for: Dataset: local government mask orders preceding statewide orders by US states
Source: F1000Res. 2021 Jan 8;10:8. [Version 1] doi: 10.12688/f1000research.27614.1 (PMC8080973; doi:10.12688/f1000research.27614.1)
Supplement: Peer review history

# Peer review: Reviewer response for version 1

**DOI:** 10.5256/f1000research.30521.r81356

**Published:** 2021-04-28

**Authors:** Karen Lee

**Affiliations:** 1 Canadian Agency for Drugs & Technologies in Health (CADTH), Ottawa, Canada

The format for this publication is appropriate as a "data note". The research reported is timely and likely to evolve over the next few months - potentially warranting an update. Information on validation of the data directly with sources might have been helpful, but likely would require more time given other priorities with the governments.

Are sufficient details of methods and materials provided to allow replication by others?

Yes

Is the rationale for creating the dataset(s) clearly described?

Yes

Are the datasets clearly presented in a useable and accessible format?

Yes

Are the protocols appropriate and is the work technically sound?

Yes

Reviewer Expertise:

Health economics, Health policy

I confirm that I have read this submission and believe that I have an appropriate level of expertise to confirm that it is of an acceptable scientific standard.

# Peer review: Reviewer response for version 1

**DOI:** 10.5256/f1000research.30521.r82423

**Published:** 2021-04-23

**Authors:** Diana M. Zuckerman, Meg Seymour

**Affiliations:** 1 National Center for Health Research, Washington, DC, USA

This is a well-done dataset and could be very useful to other researchers. It should be accepted. Our only suggestion is that perhaps listing the dates numerically on the spread sheet would be easier to read. On the other hand, having the months listed as words might make it easier to search.

Are sufficient details of methods and materials provided to allow replication by others?

Yes

Is the rationale for creating the dataset(s) clearly described?

Yes

Are the datasets clearly presented in a useable and accessible format?

Yes

Are the protocols appropriate and is the work technically sound?

Yes

Reviewer Expertise:

epidemiology

We confirm that we have read this submission and believe that we have an appropriate level of expertise to confirm that it is of an acceptable scientific standard.

# Peer review: Reviewer response for version 1

**DOI:** 10.5256/f1000research.30521.r77018

**Published:** 2021-01-26

**Authors:** Ying-chu NG

**Affiliations:** 1 Hong Kong Baptist University, Hong Kong, China

No further comments. The study seems to be done in an appropriate manner. The issue addressed in the article is timely needed in these days. Any studies about commenting on government policy on preventing COVID-19 can make use of the data for analysis. With persistence of COVID-19, the data can be updated in the coming months.

Are sufficient details of methods and materials provided to allow replication by others?

Yes

Is the rationale for creating the dataset(s) clearly described?

Yes

Are the datasets clearly presented in a useable and accessible format?

Yes

Are the protocols appropriate and is the work technically sound?

Yes

Reviewer Expertise:

health care services research

I confirm that I have read this submission and believe that I have an appropriate level of expertise to confirm that it is of an acceptable scientific standard.
